# Organ-Protective Effects and the Underlying Mechanism of Dexmedetomidine

**DOI:** 10.1155/2020/6136105

**Published:** 2020-05-09

**Authors:** Naren Bao, Bing Tang

**Affiliations:** Department of Anesthesiology, The First Hospital of China Medical University, 155 Nanjing North Street, Shenyang, China

## Abstract

Dexmedetomidine (DEX) is a highly selective *α*2 adrenergic receptor (*α*2AR) agonist currently used in clinical settings. Because DEX has dose-dependent advantages of sedation, analgesia, antianxiety, inhibition of sympathetic nervous system activity, cardiovascular stabilization, and significant reduction of postoperative delirium and agitation, but does not produce respiratory depression and agitation, it is widely used in clinical anesthesia and ICU departments. In recent years, much clinical study and basic research has confirmed that DEX has a protective effect on a variety of organs, including the nervous system, heart, lungs, kidneys, liver, and small intestine. It acts by reducing the inflammatory response in these organs, activating antiapoptotic signaling pathways which protect cells from damage. Therefore, based on wide clinical application and safety, DEX may become a promising clinical multiorgan protection drug in the future. In this article, we review the physiological effects related to organ protection in *α*2AR agonists along with the organ-protective effects and mechanisms of DEX to understand their combined application value.

## 1. Introduction

Dexmedetomidine (DEX) is a highly selective *α*2 adrenergic receptor (*α*2AR) agonist currently used in the clinic which binds in ratio to the adrenergic receptor—*α*2 : *α*1 is close to 1620 : 1. In 1999, the United States Drug and Food Administration (FDA) approved DEX for sedation and analgesia in short-term intensive care [1]. In 2008, DEX was approved by the FDA for pre- and intraoperative sedation in nontracheal intubation patients. It was then approved, in 2009, for sedating patients under general anesthesia, endotracheal intubation, and mechanical ventilation. Because DEX has the dose-dependent advantages of sedation, analgesia, antianxiety, inhibition of sympathetic nervous system activity, cardiovascular stabilization, and significant reduction of postoperative delirium and agitation, but does not produce respiratory depression and agitation, it is widely used in clinical anesthesia and the ICU.

In recent years, much clinical and basic research has confirmed that DEX has a protective effect on a variety of organs, including the nervous system, heart, lungs, kidneys, liver, and small intestine. It reduces inflammatory response, activating antiapoptotic signaling pathways which protect cells from damage. Therefore, based on wide clinical application and safety, DEX may become a promising clinical multiorgan protection drug in the future. In this article, we review the physiological effects related to organ protection in *α*2AR agonists along with the organ-protective effects and mechanisms of DEX to understand their combined application value.

## 2. Main Physiological Effects and Molecular Mechanisms Related to Organ Protection by *α*2AR Agonists

In the body, *α*2 adrenoceptors (*α*2AR) have three subtypes including *α*2A, *α*2B, and *α*2C. These are widely distributed in the central and peripheral nervous systems, autonomic ganglia, vital organs, and blood vessels [2, 3]. The main physiological effects and molecular mechanisms involved in organ protection by *α*2ARs are summarized as follows.

### 2.1. Sedative and Hypnotic Effects

Central norepinephrine has the effect of maintaining brain arousal. *α*2AR agonists activate the *α*2AR located in the locus coeruleus of the brainstem, producing an inhibition of adenylate cyclase activity and reduction of cyclic adenosine monophosphate. They also promote the outflow of potassium ions and inhibit the inflow of calcium ions in nerve endings, which leads to hyperpolarization of the cell membrane. This inhibits the discharge of neurons in the locus coeruleus and the release of norepinephrine. All these effects produce sedation and hypnosis [4].

### 2.2. Analgesic Effect

*α*2AR agonists produce analgesic effects in multiple sites. In the brain, they bind to the *α*2AR in the brainstem locus coeruleus to stop the transmission of pain signals. In the spinal cord, they activate *α*2AR in the presynaptic membranes of neurons in the posterior horn and the postsynaptic membranes of intermediate neurons to open the potassium ion channel. Thus, promotion of potassium ion outflow and inhibition of calcium ion inflow lead to the cell membrane's hyperpolarization, ultimately inhibiting the transmission of pain signals to the brain. In the periphery, they inhibit the activation of nociceptive neurons by A*δ* and C-type nerve fibers along with the release of the nociceptive neurotransmitter P and other nociceptive peptides [5].

### 2.3. Antisympathetic Effects

*α*2AR agonists can activate the *α*2AR of the brainstem locus coeruleus to inhibit the release of norepinephrine through negative feedback. This can inhibit sympathetic nerve excitability, reduce plasma catecholamine concentration, stabilize hemodynamics, reduce blood pressure and heart rate, and produce antisympathetic effects [6].

### 2.4. Affects G Protein and Intracellular Signaling

*α*2ARs belong to the G protein-coupled receptors (GPCRs) across the cell membrane and have the general biological properties of G protein. GPCRs are extensively expressed throughout the body. When they are activated, GPCRs interact with their cognate G protein. They transmit extracellular stimulation into the cell by amplifying transduction and triggering a series of intracellular responses. G proteins, being heterotrimeric, are composed of three subunits: *α*, *β*, and *γ*. The enormous quantities of G protein families—e.g., 18*α* subunits, 12*β* subunits, and 5*γ* subunits—indicate that the signal transduction pathway triggered by GPCR is very intricate [7]. The G proteins activating adenylate cyclase are called Gs, and other inhibitors are called Gi. *α*2ARs are classic inhibitory GPCRs [8, 9] which couple to the pertussis toxin-sensitive inhibitory G protein (G*α*i) and produce a multifarious biological effect in multiple organs. However, the molecular mechanism after *α*2AR receptor activation is not clear. At present, some believe that *α*2AR directly inhibits the AC-cAMP-PKA pathway through G*α*i proteins and the phosphorylation of the cAMP response element-binding protein (CREB) [10, 11]. Additionally, the separation of the G*βγ* subunit from G*α*i activates the PI3K/Akt pathway, phospholipase C, ERK, etc. [9, 12–14]. Both of the pathways mentioned above ultimately lead to the activation of NF-*κ*B [9]. However, some others point out that *α*2ARs can couple both physically and functionally to Gs, producing diametrically opposite physiological effects under high concentrations of agonist and high receptor expression. Moreover, this Gs coupling is subtype-selective [15, 16]. In fact, it seems to be inconsistent with the physiological effects of *α*2AR as a GPCR; most clinical researchers show that DEX produces anti-inflammatory and organ-protective effects by inhibiting NF-*κ*B. Based on the results of the above discussion, we speculate that DEX may couple with Gs to stimulate AC-cAMP-PKA pathways or affect PI3K/Akt pathways, etc., thus inhibiting NF-*κ*B in situations such as inflammation and ischemia, or applications within a certain concentration range.

### 2.5. Inhibits the Activity of Adenylyl Cyclase (AC)

**Figure 1:**
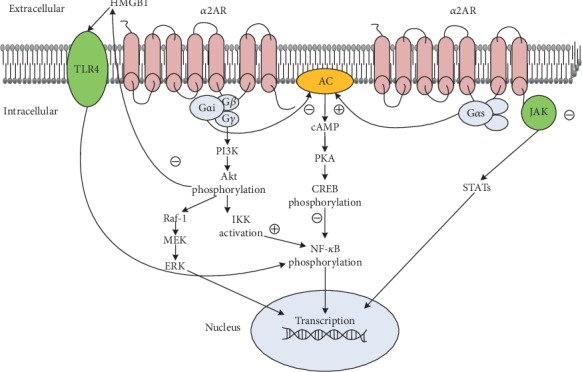
Main mechanisms of the organ-protective effects of *α*2AR agonists. *α*2ARs are classic inhibitory GPCRs (Gi). On the one hand, *α*2ARs directly inhibit the AC-cAMP-PKA pathway through G*α*i proteins; on the other hand, the separation of the G*βγ* subunit from G*α*i activates the PI3K/Akt pathway and influences a series of downstream signaling including MEK/ERK, HGMB1/TLR4/NF-*κ*B, and IKK/NF-*κ*B. Incidentally, *α*2AR agonists may inhibit JAK/STAT pathways. But when under high concentrations of an agonist and high receptor expression, *α*2ARs may couple both physically and functionally to Gs, producing an opposite effect of inhibiting NF-*κ*B.

Cyclic adenosine monophosphate (cAMP), an important intracellular functional regulator, regulates the target protein activity by controlling its phosphorylation. AC is the main factor in regulating the amount of intracellular cAMP, and a change in its activity directly leads to cAMP synthesis change. Studies have pointed out that agonistic *α*2AR has the ability to inhibit AC activity in order to reduce intracellular cAMP [17]. However, others have suggested that low-dose *α*2AR agonists reduce cAMP levels by inhibiting AC activity and vice versa. Pohjanoksa and his colleagues believe that *α*2AR-related AC regulation depends on the *α*2AR density and the activity of AC at the time [18]. Thus, it can be seen that the effect of *α*2AR agonists on the activity of AC has a subtle dual-direction regulation effect, which is related to the physical condition [16, 18, 19]. The main molecular mechanisms governing organ protection by *α*2AR agonists are shown in Figure 1.

## 3. The Main Biological Effects of Using Dexmedetomidine for Organ Protection

### 3.1. Anti-Inflammatory

In addition to the main biological effects of *α*2AR agonists, DEX has confirmed the anti-inflammatory effect of *α*2AR agonists by reducing the inflammatory cytokine (such as TNF-*α* and IL-6) in vitro, in vivo, and in clinical experiments [20, 21]. Since then, DEX's organ-protective effects as an anti-inflammatory have become a popular topic. In summary, the anti-inflammatory effects of DEX function through the following means: inhibition of TLR4/NF-*κ*B [13, 22–24], JAK2-STAT3 [25, 26], and NF-*κ*B/COX-2 [27] pathways. DEX also promotes the release of acetylcholine (ACh) through an antisympathetic effect; this combines with *α*7nAChR on immune cytomembranes and exerts anti-inflammatory effects via the cholinergic pathway (as in Figure 1) [13, 26, 28].

### 3.2. Antiapoptotic

With the advancement of DEX research in recent years, researchers have found that DEX also has an antiapoptotic effect. The antiapoptosis function of DEX is achieved by activating the PI3K/Akt signaling pathway [29], the Bax/cytochrome C/caspase pathway [15], and the ERK1/2 signaling pathway [18, 30], as well as inhibiting Notch/NF-*κ*B signaling pathways [31] and activating the mitochondrial ATP-sensitive K+ (mitoKATP) channels (see Figure 1) [32, 33].

## 4. The Mechanism of Organ-Protective Effects of Dexmedetomidine

### 4.1. Nerve System Protection

In studies of the protective effects of DEX on organs, those focused on the nervous system were the earliest and deepest. Jiang et al. [34] first reported the protective effect of DEX on hypoxic-ischemic brain damage in 1991. In the past 20 years, people have mainly focused on three aspects: (1) protecting against cerebral ischemia/hypoxia injury and improving nervous system function after brain injury [35], (2) reducing the damage of anesthetics on developing neurons [36–38], and (3) decreasing the incidence of postoperative delirium or cognitive dysfunction [39, 40]. Currently, great progress has been made in research on the effects and mechanisms of DEX, suggesting that DEX has application values in newborns, children, patients with craniocerebral injury or stroke, and elderly patients with degraded cerebral function. Going forward, more efforts will be needed to validate laboratory research in the clinic.

Regarding the protective effect of DEX on the nervous system, it has five main functions: (1) inhibiting the excitability of sympathetic nerves and regulating the release of catecholamines, (2) regulating the release of central glutamate, (3) inhibiting cell apoptosis and release of inflammatory cytokines, (4) antioxidant stress, and (5) regulating synaptic plasticity and reducing neurotoxicity of anesthetics. The inhibition of cell apoptosis and release of inflammatory cytokines are considered to be paramount.

#### 4.1.1. Regulates the Release of Catecholamine

Cerebral ischemia/hypoxia causes the brain to superfluously release catecholamines. This causes calcium overload in neurons, generates abundant neurotoxic free radicals, aggravates ischemia and hydrocephalus through severe cerebrovascular contraction, and increases the sensitivity of neurons to glutamate, exacerbating its destructiveness [41, 42]. DEX can effectively reduce the production of catecholamine, reducing cerebral vascular spasms and subsequent brain damage [16, 35].

#### 4.1.2. Inhibits the Release of Glutamate

Glutamate, an excitatory neurotransmitter released during cerebral ischemia, excites receptors and causes neuronal damage. DEX dose-dependently inhibits the release of glutamate caused by multiple channels [43, 44], significantly reduces the hypoxia and depolarization-induced increase of extracellular glutamate [45], and reduces the accumulation of glutamate by inhibiting the absorption of neurocyte to glutamate.

#### 4.1.3. Anti-Inflammatory and Antiapoptotic Effects

Cerebral ischemia, traumatic injury, and postoperative cognitive dysfunction (POCD) are closely related to neuron inflammation and apoptosis. DEX has shown its anti-inflammatory and antiapoptotic effects in multiple animal models [46–51]. As we mentioned above, the anti-inflammatory effect of DEX is achieved by inhibiting the TLR4/NF-*κ*B [47], JAK2-STAT3 [25, 50], and NF-*κ*B/COX-2 pathways [27, 52]; activating the ERK1/2 pathway [53]; and releasing acetylcholine (ACh) through antisympathetic effects via the cholinergic pathway [13]. At the same time, DEX also reduces neuronal apoptosis through a variety of mechanisms that enhance the viability of the neurocyte. These include increasing the expression of antiapoptotic proteins Mdm-2 and Bcl-2, inhibiting proapoptotic Bax and p53, reducing the permeability of mitochondrial membranes, reducing the release of cytochrome C and apoptosis-inducing factors into the cytoplasm [54], activating the PI3K/Akt pathway and reducing cysteinyl aspartate proteinase (caspase-3) by promoting the phosphorylation of the focal adhesion kinase (FAK) [43], enhancing the phosphorylation of ERK1/2 by inhibiting neuronal sodium ions and delaying potassium ion influx [4, 55], and inhibiting the Notch/NF-*κ*B signaling pathway [31].

#### 4.1.4. Antioxidant Stress

The brain is very sensitive to the destruction of oxygen-free radicals, and ischemia-reperfusion injuries produce an intracorporal antioxidant-peroxidation state imbalance. DEX eliminates the excessive free radicals in the body and reduces this pathological chain reaction by reducing malondialdehyde (MDA) and improving the activity of superoxide dismutase [56, 57].

#### 4.1.5. Reduces Neurotoxicity of Anesthetics and Regulates Synaptic Plasticity

Many studies have confirmed that general anesthetics cause neurotoxicity in the developing brain, produce neural apoptosis, and inhibit the establishment of connections between synapses [36, 45]. Three large retrospective clinical studies showed that the general anesthesia of healthy children under 2 years of age had little correlation with postoperative neurodevelopmental hypogenesis. However, the risk in premature babies and those with congenital heart disease is greatly increased [58]. Based on this, in 2016, the FDA reiterated that repeated application of general anesthetics and sedatives to children under three years old and women in the last three months of pregnancy may affect child and fetus development.

DEX reduces the neurotoxicity of sevoflurane through many pathways: inhibiting apoptosis and autophagy [59], increasing the expression of tyrosine kinase B (TrkB) and BDNF, promoting neurocyte proliferation, maintaining nervous system function [60], inhibiting neuronal mitochondrial dynamin-related protein (Drp1) [37], and dose-dependently activating the bone morphogenetic protein (BMP)/Smad pathway to regulate self-renewal, differentiation, proliferation, migration, and apoptosis of the neurocyte [61]. The multiple signal pathways we mentioned above show that DEX has played a significant role in neuroprotection during infant development, laying the foundation for the safe adhibition of DEX in children and pregnant women and solving the problem of general anesthesia drug selection for children.

Synaptic plasticity, the property of adjustable connection between synapses, is closely related to learning and memory since it controls synaptic information transmission. DEX pretreatment significantly improved the decline of the proliferative capacity and decreased neuronal plasticity in neonatal rats after hyperoxia induction [62]. The research on regulation of synaptic plasticity through DEX has important implications for neurodevelopmental protection and promotes the study of synaptic plasticity regulation. What is more, it is a meaningful breakthrough in the treatment of neurological diseases such as memory deficits.

### 4.2. Cardioprotective Benefits

Intraoperative patients are susceptible to stimuli such as surgical procedures and endotracheal intubation, which cause excitation in sympathetic nerves. This can lead to tachycardia, increased blood pressure, imbalance of myocardial blood oxygen supply and demand, and cardiac complications. The main pharmacological effects of DEX are reducing the excitability of the sympathetic nervous system, weakening the stress response, and stabilizing hemodynamics. Thus, it effectively prevents the occurrence of myocardial ischemia in the perioperative period. A large number of clinical observations have confirmed that DEX reduces postoperative mortality and myocardial infarction [63, 64].

We conclude that the cardioprotective mechanism of DEX is mainly reflected in the following three aspects:

1. Inhibition of norepinephrine neuron activity in the locus coeruleus, which suppresses sympathetic nerve excitation to reduce catecholamine levels in the blood, cardiac load, and myocardial oxygen consumption. At the same time, it prolongs diastolic perfusion time, increases left ventricular coronary blood flow, reduces myocardial lactic acid release, and improves myocardial resistance to ischemia and hypoxia
2. DEX directly inhibits cardiac norepinephrine release to reduce the occurrence of arrhythmias in high-risk patients
3. Anti-inflammation and antiapoptosis: DEX pretreatment activates some of the signal pathways through G protein like PI3K/Akt and MEK1-2-ERK1/2, sequentially reducing apoptosis and inflammatory response caused by ischemia-reperfusion and reducing the myocardial infarction area [65–67]. The downstream molecular mechanism is not completely clear at present

Through a review of laboratory and clinical research, a new perspective is gradually formed: rational use of DEX during the perioperative period may effectively reduce the risk of cardiovascular surgery [68, 69]. However, experiments by Mimuro et al. [70] have questioned this. They found that during ischemia-reperfusion injury in mouse myocardium, DEX increases the myocardial infarction area without altering hemodynamics and coronary blood flow. This effect is antagonized by yohimbine, an *α*2AR antagonist. Although such objections are in the minority, they still need to be taken seriously. Therefore, the cardioprotective effects of DEX need to be further studied and discussed.

### 4.3. Pulmonary Protection

The lungs, as sensitive organs, are extremely vulnerable to systemic inflammation and remote organ ischemia-reperfusion injury. Many clinical practices—such as trauma, one-lung ventilation, extracorporeal circulation, and liver transplantation—cause lung damage through inflammation and apoptosis. Ventilation-associated lung injury (VALI) is also frequently associated with acute respiratory distress syndrome (ARDS) during treatment. In recent years, it has been observed that DEX has pulmonary protection benefits in many cases of lung injuries through its effect on the pulmonary vasoconstriction mechanism, pulmonary vascular ischemia-reperfusion injury, and release of inflammatory cytokine.

#### 4.3.1. Relieves Inflammation, Apoptosis, and Oxidative Stress from Various Pathological Injuries

In models of acute lung injury caused by renal ischemia-reperfusion injury, DEX pre- and posttreatment significantly reduce pulmonary edema and inflammatory response by reducing myeloperoxidase (MPO) activity as well as downregulating intercellular adhesion molecule-1 (ICAM-1) and TNF-*α* mRNA expression [71, 72]. In the rat sepsis model [24], DEX inhibits inflammatory response in the lung by the TLR4/myeloid differentiation factor 88/NF-*κ*B pathway and reduces mortality by activating PI3K/Akt/mTOR pathways. In the rat chest trauma model, DEX also has a protective effect on pulmonary contusion [73], which reduces proinflammatory cytokines by inhibiting the activity of NF-*κ*B. Furthermore, DEX reduces mitochondrial dysfunction, oxidative stress, and apoptosis in LPS-induced acute lung injury [74]. Moreover, the pulmonary protection effect of DEX has a parabolic correlation with its concentration. It shows that 50 *μ*g/kg is the strongest concentration: 10 and 100 *μ*g/kg did have an effect, but it was weaker than at 50 *μ*g/kg [54, 75].

#### 4.3.2. Reduces VALI and Hypoxic Pulmonary Vasoconstriction

On the one hand, VALI is caused by diffuse alveolocapillary membrane damage and increased permeability due to excessive airway pressure (volutrauma). On the other hand, intense mechanical stretching activates a variety of mediators involved in inflammation in the lung's inherent cells, promotes the transcription and expression of proinflammatory factors, triggers a waterfall of inflammation, and eventually leads to the occurrence of lung injury [76]. Yang et al. [77] used the high tidal volume ventilation (HVT) mode to induce lung injury in rats. When they intravenously injected ten times the clinical dose, DEX significantly improved the lung injury with respect to its pathological morphology, inflammatory cytokines, and chemokines.

In addition, DEX improves hypoxic pulmonary vasoconstriction (HPV) and oxygenation during one-lung ventilation. In an isolated lung, the imbalance of ventilator-flow ratios causes hypoxemia; in response, the body compensates for HPV by reducing the abnormal distribution of pulmonary blood flow. During anesthesia, HPV is often affected by anesthetics such as isoflurane, sevoflurane, and propofol [78]. The application of DEX indirectly affects HPV by reducing the medicinal dose of inhalation and intravenous anesthesia. At the same time [79], DEX directly enhances HPV and alters oxygenation by reducing oxidative stress and increasing nitric oxide release during one-lung ventilation.

### 4.4. Renal Protection

The renal blood supply accounts for 20% of cardiac output, and 90%–95% of this supply is distributed in the cortex. The perioperative shock, extracorporeal circulation, and usage of vasoactive drugs can easily lead to ischemia-reperfusion injury (I/R) in the renal parenchyma, especially the cortex [26, 80]. The renoprotective effects of DEX are as follows.

#### 4.4.1. Improves Local Renal Blood Flow and Diuresis

Ischemia-reperfusion leads to an increase in systemic and local sympathetic activity along with intense vasoconstriction in the renal cortex. DEX improves renal ischemia damage by improving outer renal medullary blood flow through local renal vasodilation [75], increasing glomerular filtration, dampening the ability of arginine vasopressin in the collecting duct, inhibiting the expression of aquaporins along with the transport of Na^+^ and water [81], urination stimulation, and so on [82]. At the same time, DEX also reduces glomerular congestion, swelling in renal tubular epithelial cells, and luminal stenosis [83]. What is more, DEX has multiple effects during the perioperative period: in addition to analgesia, it reduces the accumulation of other analgesic and nonsteroidal anti-inflammatory drugs that increase kidney injury risk.

#### 4.4.2. Inhibits Inflammation, Oxidative Stress, and Apoptosis

As we mentioned above, DEX blocks NF-*κ*B transcription by inhibiting the TLR4/NF-*κ*B and JAK/STAT pathways while activating *α*7nAChR via the cholinergic pathway, thus producing anti-inflammatory effects [13, 26, 84, 85]. As a synthetic iNOS promoter, obstruction of NF-*κ*B may reduce NO release, prevent oxidative stress, and reduce mitochondrial damage [86]. DEX also reduces oxidative stress by promoting the Keapl/Nrf2/ARE/HO-1 pathway [87]. Chen et al. [88] proposed that DEX exerts antiapoptotic functions by inhibiting the ROS/JNK signaling pathway, thus reducing the expression of Bax, cytochrome C, cleaved caspase-9 proteins, and cleaved caspase-3 proteins in the mitochondrial-dependent pathway.

#### 4.4.3. Relieves Hypercoagulability

When sepsis or ischemia-reperfusion injury occurs, various inflammatory cytokines stimulate endotheliocytes to release inflammatory mediators and procoagulant substances. This activates the coagulation system, leading to a hypercoagulability state. Thereafter, the diffuse microthrombus is formed in the glomerulus (which causes perirenal blood stasis), and the glomerular filtration rate decreases. The inflammatory response interacts with the abnormal coagulation, causing accumulation of the toxic and harmful metabolites, aggravating kidney damage, and ultimately leading to acute and chronic kidney disease. The antisympathetic effect of DEX weakens the stress response, thereby relieving the hypercoagulable state [89]. In addition, DEX can dilate blood vessels, accelerate blood flow, reduce erythrocyte aggregation, reverse blood stagnation, and reduce kidney damage caused by coagulopathy.

### 4.5. Hepatoprotective Effects

DEX also has hepatoprotective benefits due to its effects on inflammation, apoptosis, and oxidative stress. In animal experiments of hepatic ischemia reperfusion injury, intraperitoneal injection of DEX (10 or100 *μ*g/kg) 30 minutes before liver ischemia can increase the levels of superoxide dismutase, catalase, and glutathione to reduce liver tissue damage [90]. In lipopolysaccharide- (LPS-) induced oxidative stress and apoptosis experiments on rat liver, it was confirmed that DEX reduces symptoms by enhancing the GSK-3*β*/MKP-1/Nrf2 pathway activity through *α*2AR [91]. Wang et al. found that DEX protects rat liver from ischemia-reperfusion injury in relation to the TLR4/NF-*κ*B pathway [33]. In the LPS/D-galactosamine-induced mouse acute liver injury experiments by Yang et al., DEX inhibited the release of TNF-*α*, phosphorylation of c-jun-N-terminal kinase (JNK), and cleavage of caspase-3. By reducing the activity of caspase-3, caspase-8, and caspase-9, it helped relieve hepatocyte apoptosis [92]. But the molecular mechanism and clinical significance of this activity require further research and clarification.

### 4.6. Intestinal Protection

The intestinal barriers are important for the body's resistance to external pathogens and toxins, inflammation, stress, surgical trauma, and hypovolemia; however, ischemia-reperfusion injury can cause intestinal barrier damage [93, 94]. Thus, bacteria and toxins in the intestine can reach the mesenteric lymphoid tissues, lymph fluid, blood, and extraintestinal tissues and organs, causing gut-derived sepsis and eventually leading to multiple organ dysfunction syndrome (MODS) [95]. Attention to the protection of intestinal barrier functions under pathological conditions can reduce the incidence of gut-derived sepsis, improve the prognosis of patients, and reduce the mortality of critical patients. Therefore, in recent years, the protection of intestinal health during the perioperative period has been increasingly recognized.

#### 4.6.1. Anti-Inflammation, Antioxidative Stress, and Antiapoptosis

The effects of DEX against inflammation, apoptosis, and oxidative stress that we have described above also function in intestinal protection. Zhang et al. [54] studied intestinal ischemia-reperfusion injuries in rats, administering DEX at different times and doses. They showed that DEX pretreatment was dose-dependent for resisting intestinal ischemia-reperfusion injury in a certain range. Their experiments suggested that in addition to the reduction of oxidative stress, inflammation indicators of malondialdehyde (MDA) and myeloperoxidase (MPO), serum levels of diamine oxidase (DAO), activity of caspase-3, ileal mucosa levels, and the apoptotic index of ileal mucosal cells were significantly lower than in those rats without DEX. It was shown that the effect of DEX is maximal at 5 *μ*g/kg/h, but has no effect in posttreatment. The mechanism of the difference in pre- and posttreatment needs further research.

In addition, some clinical trials have also highlighted the intestinal protective effect of DEX. DEX pretreatment relieves intestinal and liver damage in patients undergoing liver cancer resection who are afflicted by selective hepatic lobectomy and liver cirrhosis [96]. DEX also has significant preventive effects on postoperative abdominal adhesions [97], and its mechanism may relate to antioxidative stress effects and reduction of tissue inflammation.

#### 4.6.2. Promotes Recovery of Intestinal Motility, Improves Intestinal Microcirculation, and Protects against Intestinal Epithelial Barrier Disruption

DEX promotes isolated rat ileum contraction [98]. In contrast, Herbert et al. [99] found that DEX dose-dependently inhibited the enterocinesia of guinea pigs' small intestines in vitro. In healthy volunteers, DEX also inhibits gastric emptying and enterocinesia [100]. These contradictory results may be related to the physiological state of the different subjects. Under normal physiological conditions, DEX may inhibit enterogastric peristalsis via enteric neurons. On the other hand, under pathological conditions such as infection, stress, trauma, and hypovolemia, DEX benefits intestinal microcirculation perfusion, protects the gut barrier, and restores gastrointestinal motility. It does the latter by reducing inflammation and stress reactions, maintaining hemodynamic stability, relieving postoperative pain, and reducing the dosage of postoperative opioids. In rat models, DEX protects against intestinal epithelial barrier disruption by recovering the density of small blood vessels perfused in the intestinal mucosa and muscles, attenuating intestinal microcirculatory dysfunction, and inhibiting inflammatory response, thus reducing mucosal cell death and tight junctional damage [56, 101].

## 5. Correlation of Anti-Inflammatory Effects with Timing and Dose

The anti-inflammatory effect of DEX has some correlation with its dose. In sepsis rats, Taniguchi et al. [102] found that the medium dose (5 *μ*g/kg/h) and high dose (10 *μ*g/k/h) of DEX reduce plasma levels of TNF-*α* and IL-6, while the low-dose group (2.5 *μ*g/kg/h) showed no decreasing effect. Similar experimental phenomena were observed in vitro [43, 103]. Lai et al. [104] believed that the anti-inflammatory effect of DEX had a parabolic relationship with its concentration. In their in vitro experiment, LPS-induced expression of inflammatory cytokines was treated with different concentrations of DEX. The result shows that while the addition of 0.01 *μ*mol/L of DEX had no impact, and the addition of 1 *μ*mol/L of DEX was significantly inhibited, 100 *μ*mol/L of DEX promoted the expression of nitric oxide synthase and nitric oxide synthesis; the effects of inflammatory cytokines such as prostaglandin E2, TNF-*α*, IL-1*β*, IL-6, and IL-10 are consistent with the effects of nitric oxide. Some other scholars have also observed the same trend [54, 55].

In addition, Gu et al. believe that the protective effect of DEX on organs is related to the processing time, and the posttreatment conditioning is less than the pretreatment [11]. Zhang et al. believe that DEX posttreatment does not alleviate dilated reperfusion injury in the small intestine [54]. Based on the physiological characteristics of *α*2AR described above, *α*2AR-related AC regulation is affected by factors such as *α*2AR density and AC activity at the time, while the latter two are related to the state of the body. So we speculate that there are two situations: (1) *α*2AR density and AC activity may be affected by different stages of inflammation in the body or organs; (2) although DEX is mainly coupled with the Gi protein, in a certain concentration range, it may also be coupled with the Gs protein. The latter leads to a subtle two-way regulation effect on AC activity and even the downstream pathways. As a result, some scholars have observed dose-dependent changes in organ protection and differential results in pre- and posttreatment. Consequently, in the future, it is necessary to search for a more safe and effective concentration range and medication timing in the usage of DEX.

## 6. Perspective

In conclusion, the organ-protective effect of DEX is achieved in different pathological conditions by multiple mechanisms. However, a majority of the current DEX studies are based on animal experiments, and the mechanism is not fully elucidated. More intensive research and clinical trials are necessary for further verification. In addition, despite the mainstream belief that the organ-protective effect of DEX seems clear, there are conflicting conclusions about whether *α*2AR agonism is protective or destructive. Therefore, while considering the sedative, analgesic, and organ-protective effects of DEX, we need to explore the different rates of timing, appropriate dosages, and medication profiles of this substance. This will help to promote realistic, significant clinical observations that validate the potential for using DEX in the future to protect organs.
